# Natural killer cells-related immune traits and amyotrophic lateral sclerosis: A Mendelian randomization study

**DOI:** 10.3389/fnins.2022.981371

**Published:** 2022-09-29

**Authors:** Zhenxiang Gong, Yang Liu, Fengfei Ding, Li Ba, Min Zhang

**Affiliations:** ^1^Department of Neurology and Psychiatry, Tongji Hospital, Tongji Medical College, Huazhong University of Science and Technology, Wuhan, China; ^2^Department of Pharmacology, School of Basic Medical Sciences, Fudan University, Shanghai, China

**Keywords:** amyotrophic lateral sclerosis (ALS), innate immunity, natural killer cells, Mendelian randomization (MR), genome-wide association study (GWAS)

## Abstract

**Background:**

Observational studies have suggested that peripheral immune disorders are associated with amyotrophic lateral sclerosis (ALS). Previous studies predominantly focused on changes in adaptive immunity. However, emerging evidence showed natural killer (NK) cells, an essential component of innate immunity, were involved in the degeneration of motor neurons. However, the causal relationship between dysregulated NK cells-related immune traits and ALS remains unclear.

**Objective:**

This study aimed to explore the causal relationship between NK cells-related immune traits and the risk of ALS.

**Materials and methods:**

Single nucleotide polymorphisms (SNPs) significantly associated with NK cells-related immune traits were selected as instrumental variables to estimate their causal effects on ALS. SNPs from a genome-wide association study (GWAS) on NK cells-related immune traits were used as exposure instruments, including an absolute NK-cells count, absolute HLA-DR^+^ NK-cells count, NK cells/lymphocytes, NK cells/CD3^–^ lymphocytes, HLA DR^+^ NK cells/NK cells, HLA DR^+^ NK cells/CD3^–^ lymphocytes, and the median fluorescence intensities of CD16^–^CD56^+^ on NK cells and HLA-DR^+^ NK cells. Summary-level GWAS statistics of ALS were used as the outcome data. Exposure and outcome data were analyzed using the two-sample Mendelian randomization (MR) method.

**Results:**

Each one standard deviation increase in the expression levels of CD16^–^CD56^+^ on NK cells and HLA-DR^+^ NK cells were associated with a lower risk of ALS in both the MR-Egger and inverse variance weighted methods (*P* < 0.05). The results proved robust under all sensitivity analyses. Neither instrumental outliers nor heterogeneity were detected.

**Conclusion:**

Our results suggest that higher expression levels of CD16^–^CD56^+^ on NK cells and HLA-DR^+^ NK cells are associated with a lower risk of ALS.

## Introduction

Amyotrophic lateral sclerosis (ALS) is a fatal neurodegenerative disorder characterized by progressive degeneration of the upper and lower motor neurons (Kiernan et al., 2011). Riluzole and edaravone are currently the most widely used medicines for ALS, and they potentially prolong survival by 3–6 months in most patients with ALS (Hardiman et al., 2011; Rothstein, 2017). Although significant effort has been devoted to investigating the pathological mechanisms underlying ALS, with several theories having been proposed, the etiology of ALS remains poorly understood (Geevasinga et al., 2016).

Accumulating evidence implicates neuroimmune crosstalk between the peripheral immune system (PIS) and central immune system (CIS) in ALS, cooperatively contributing to the death of motor neurons (Yu et al., 2022). An increased infiltration of peripheral immune cells into the spinal cord and motor cortex of patients with ALS has been observed, which, together with activated microglia and astrocytes, constitute a pro-inflammatory immune microenvironment (Beers and Appel, 2019). Previous studies have linked changes in the PIS with disease progression in both mouse models and patients with ALS (Zhao et al., 2013; Murdock et al., 2016; McCombe et al., 2020). Specifically, the total leukocytes count is elevated in patients with ALS, and the count of anti-inflammatory regulatory T cells (Tregs) negatively correlates with disease progression (Beers et al., 2017; Sheean et al., 2018). These studies have indicated that peripheral immune disorders and systemic inflammation are associated with ALS. In addition to previous reports on changes in adaptive immunity, a considerable number of studies have recently reported innate immune disorders in ALS, providing insight into the novel pathological mechanisms of ALS.

As an important component of innate immune cells, natural killer (NK) cells are important components of the first line of immune defense in humans and essential regulators of adaptive immunity (Abel et al., 2018). Conventionally, human NK cells are defined by the expression of CD56 and lack of CD3 (Cooper et al., 2001a). Preliminary studies have demonstrated that NK cells may be involved in the development of ALS. Under normal physiological conditions, motor neurons are protected from NK-cells damage by the expression of human leukocyte antigen A–C surface markers, which are lost in spinal cord motor neurons in ALS due to unknown mechanisms, rendering them vulnerable to NK-cells damage (Song et al., 2016). Garofalo et al. (2020) revealed that NK cells in the spinal cord instruct microglia toward an inflammatory phenotype in an interferon-γ (IFN-γ)-dependent manner in a mouse model of ALS (hSOD1^*G*93*A*^). However, it is important to note that not all NK cells are prolific IFN-γ producers, and the functions of NK cells are closely related to the expression of surface markers, such as CD16, CD56, CD57, and CD161. Compared with other subsets of NK cells, CD16^–^CD56^+^ NK cells and HLA-DR^+^ NK cells are two functionally activated subsets associated with high IFN-γ production (Freud et al., 2017; Erokhina et al., 2018, 2021). The currently limited understanding of NK cells in ALS may be due to insufficient discrimination of NK cells-related immune traits in previous studies. Therefore, we emphasize that distinguishing NK cells subsets is an important step in exploring the role of NK cells in ALS.

In medical research, conclusions drawn from observational studies are susceptible to methodological inconsistencies, confounding factors, and selection bias (Sedgwick, 2015). Randomized controlled trials (RCTs) are a promising tool for making causal inferences; however, the implementation of RCTs requires tremendous human and financial effort and is occasionally subject to sample size and ethical issues, especially in the field of rare diseases, such as ALS. Mendelian randomization (MR) is an emerging approach for assessing the causal relationship between exposure and outcome (Emdin et al., 2017). Based on Mendel’s law of inheritance regarding the random assignment of parental alleles to offspring, the MR approach can be considered a “natural” RCT, capable of reducing the conventional bias of observational studies and compensating for the inadequacy of RCTs in investigating rare diseases (Emdin et al., 2017). Therefore, to evaluate the causal relationship between NK cells-related immune traits and ALS, we conducted a MR study. By leveraging the single nucleotide polymorphism (SNP) data from a large genome-wide association study (GWAS) on immune-cells traits and summary-GWAS ALS statistics, we found a higher expression of CD16^–^CD56^+^ on NK cells and HLA-DR^+^ NK cells to be causally associated with a lower risk of ALS.

## Materials and methods

### Exposure data and instrumental variable selection

The appropriate genetic variants were selected from a previous GWAS study that assessed the impact of natural genetic variation on immune-cells traits (Orrù et al., 2020). In the current study, we obtained summary-level association results for eight independent NK cells-related immune traits, including the absolute counts of NK cells and HLA-DR^+^ NK cells; ratios of NK cells/lymphocytes, NK cells/CD3^–^ lymphocytes, HLA-DR^+^ NK cells/NK cells, and HLA-DR^+^ NK cells/CD3^–^ lymphocytes; and the median fluorescence intensity (MFI) of CD16^–^CD56^+^ on NK cells and HLA-DR^+^ NK cells. The genetic IVs, SNPs in this case, were filtered according to a previously described procedure (Xia et al., 2022). Briefly, SNPs associated with NK cells-related immune traits were extracted as IVs at a genome-wide significance level (*P* < 5.00E-8). Second, the extracted IVs were subsequently clumped based on the 1000 Genomes Project linkage disequilibrium structure (R^2^ < 0.001), and only the SNPs with the lowest *P*-values were retained (Abecasis et al., 2012). Third, certain SNPs that were not available in the summary statistics of the outcome were replaced with proxy SNPs with a high correlation coefficient (*R*^2^ ≥ 0.8) based on European ancestry. Fourth, F-statistics for each SNP were calculated to quantify the strength of the instruments, and weak SNPs were excluded (*F* < 10) (Burgess et al., 2016). Summarized information pertaining to all the IVs included in this study is shown in the Supplementary Table.

### Outcome data

Publicly available GWAS summary statistics for ALS were obtained from a previous study involving a sample of 80,810 individuals of European ancestry (20,806 ALS cases and 59,804 control cases) (Nicolas et al., 2018). Kinesin family member 5A (*KIF5A*) was identified as a novel gene associated with ALS in this GWAS study. All patients with ALS in this GWAS study had been diagnosed with ALS at probable or definite levels according to the El Escorial criteria (Brooks, 1994) and had experienced symptom onset after 18 years of age. The outcome and exposure data were harmonized to exclude strand mismatches and ensure the alignment of SNP effect sizes (Burgess et al., 2019).

### Two-sample Mendelian randomization

**FIGURE 1:**
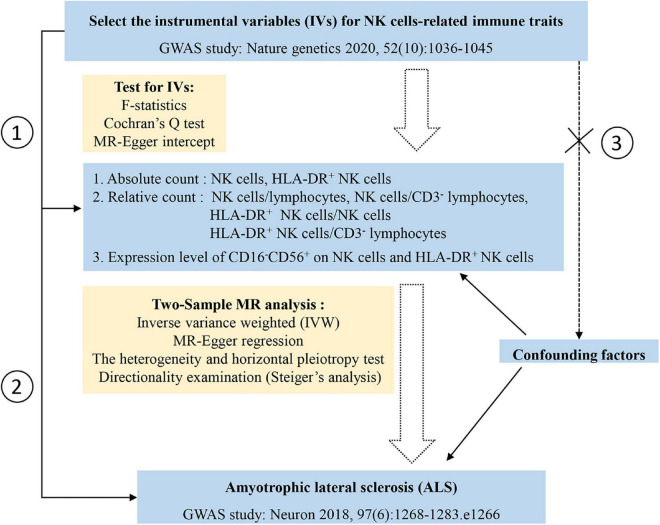
Study design and the flow chart of statistical analysis. Three assumptions of the Mendelian randomization in the current study: assumption 1, the selected genetic variances, as instrumental variables, are associated with the risk factor, that is, natural killer (NK) cells-related immune traits in this case; assumption 2, the selected genetic variances are significantly associated with amyotrophic lateral sclerosis (ALS) through the risk factor only; assumption 3, the selected instrumental variables are not associated other confounding factors (the dashed line and cross symbol indicate no association between selected instrumental variables and confounding factors).

The theoretical basis of two-sample MR research relies on three core assumptions: (1) the selected genetic variances, as IVs, are associated with the risk factor, that is, NK cells-related immune traits in this case; (2) the selected genetic variances are significantly associated with ALS through the risk factor only; and (3) the selected IVs are not associated with other confounding factors. In MR analysis, when the core assumptions are satisfied, the inverse variance weighted (IVW) method can improve the statistical power and accuracy of the estimation (Burgess et al., 2017); therefore, the IVW method was implemented as the main approach to examining the overall causal relationship between NK cells-related immune traits and ALS based on the effect of IVs. However, when horizontal pleiotropy exists, the causal estimate using the IVW method is potentially biased. Thus, genetic variants may affect ALS susceptibility through pathways other than NK cells-related immune traits. The presence of pleiotropy was detected when the intercept significantly deviated from the origin in the MR-Egger regression method (Hemani et al., 2018). MR-Egger regression and weighted median was implemented as complementary methods to examine the effect of NK cells-related immune traits on ALS. We conducted Cochran’s Q test and leave-one-SNP-out analysis using the IVW method to evaluate the heterogeneity of the IVs. Steiger’s analysis was used to explore whether ALS had a causal impact on NK cells-related immune traits (Hemani et al., 2017). A flow diagram depicting the study process is shown in Figure 1. All analyses were performed using the TwoSampleMR and RadialMR packages in R software (version 4.2.0).

## Results

**FIGURE 2:**
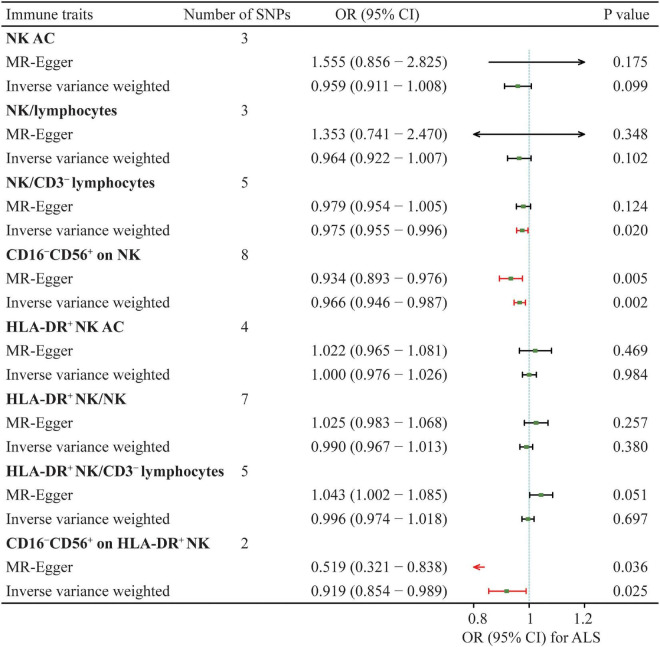
Summary of the causal effects of NK cells-related immune traits on ALS with mendelian randomization. ALS, amyotrophic lateral sclerosis; SNP, single nucleotide polymorphism; NK, natural killer cells; HLA, human leucocyte antigen; OR, odds ratio; CI, confidence interval.

**FIGURE 3:**
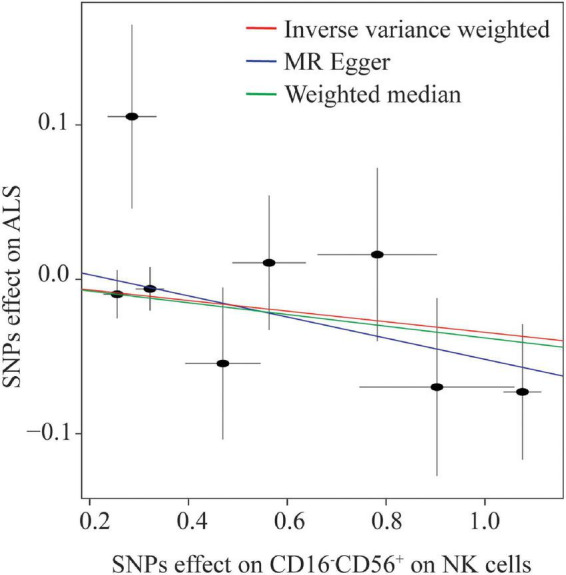
Scatterplot of single nucleotide polymorphism (SNP) effects on the expression level of CD16^–^CD56^+^ on natural killer (NK) cells versus amyotrophic lateral sclerosis (ALS), with the slope of each line corresponding to the estimated Mendelian randomization effect per method.

Eight NK cells-related immune traits were analyzed for their association with ALS in the current study. The association statistics are summarized in Table 1 and Figure 2. The results revealed that a one standard deviation (1-SD) increase in the MFI of CD16^–^CD56^+^ on NK cells was associated with a lower risk of ALS in both the IVW method (odds ratio [OR] = 0.966, 95% confidence interval [CI] = 0.946–0.987, *P* = 0.002) and MR-Egger (OR = 0.934, 95% CI = 0.893–0.976, *P* = 0.005; Figure 3). The MFI of CD16^–^CD56^+^ on HLA-DR^+^ NK cells was also negatively associated with the risk of ALS in the IVW method (OR = 0.919, 95% CI = 0.854–0.989, *P* = 0.025) and MR-Egger (OR = 0.519, 95% CI = 0.321–0.838, *P* = 0.036; Supplementary Figure 1). The increased ratio of NK cells/CD3^–^ lymphocytes was negatively associated with the risk of ALS in the IVW method (OR = 0.975, 95% CI = 0.955–0.996, *P* = 0.020) but not in the MR-Egger method (OR = 0.979, 95% CI = 0.954–1.005, *P* = 0.124).

**TABLE 1:** Summary of the causal effects of natural killer (NK) cells-related immune traits on amyotrophic lateral sclerosis (ALS) with various Mendelian randomization (MR) methods.

Immune traits	Inverse variance weighted		MR-Egger		Cochran’s Q	MR-Egger	
	OR (95% CI)	*P*-value	OR (95% CI)	*P*-value		Intercept	*P*-value
NK AC	0.959 (0.911–1.008)	0.099	1.555 (0.856–2.825)	0.175	3.101	−0.099	0.139
NK/lymphocytes	0.964 (0.922–1.007)	0.102	1.353 (0.741–2.470)	0.348	3.004	−0.082	0.294
NK/CD3^–^ lymphocytes	0.975 (0.955–0.996)	0.020	0.979 (0.954–1.005)	0.124	3.191	−0.003	0.608
CD16^–^CD56^+^ on NK	0.966 (0.946–0.987)	0.002	0.934 (0.893–0.976)	0.005	30.501	0.017	0.094
HLA-DR^+^ NK AC	1.000 (0.976–1.026)	0.984	1.022 (0.965–1.081)	0.469	4.951	−0.010	0.427
HLA-DR^+^ NK/NK	0.990 (0.967–1.013)	0.380	1.025 (0.983–1.068)	0.257	20.019	−0.011	0.056
HLA-DR^+^ NK/CD3^–^ lymphocytes	0.996 (0.974–1.018)	0.697	1.043 (1.002–1.085)	0.051	18.560	−0.019	0.013
CD16^–^CD56^+^ on HLA-DR^+^ NK	0.919 (0.854–0.989)	0.025	0.519 (0.321–0.838)	0.036	5.597	0.152	0.056

MR, Mendelian randomization; ALS, amyotrophic lateral sclerosis; NK, natural killer cells; HLA, human leukocyte antigen; OR, odds ratio; CI, confidence interval.

**FIGURE 4:**
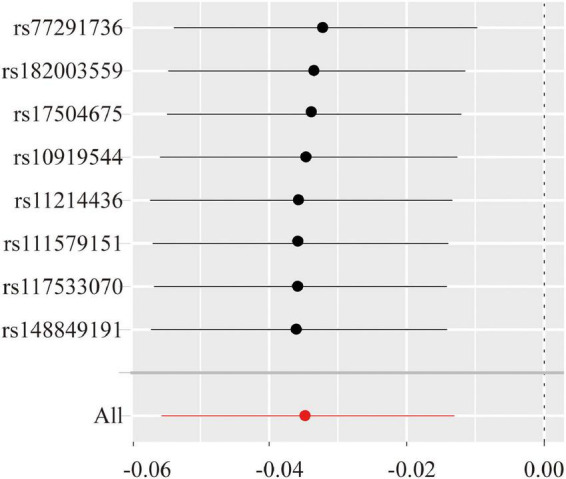
The leave-one-single nucleotide polymorphism (ANP)-out sensitivity analyze for the expression level of CD16^–^CD56^+^ on natural killer (NK) cells versus amyotrophic lateral sclerosis (ALS). The Y-axis shows the ID of the SNP to be omitted from analysis and the X-axis represents Mendelian randomization (MR) odds ratios. The red line shows MR estimate including all SNPs.

Thereafter, extensive sensitivity analyses were performed to validate the causal association between NK cells-related immune traits (CD16^–^CD56^+^ on NK cells and HLA-DR^+^ NK cells) and ALS. No heterogeneity was detected using Cochran’s Q test (Q pval > 0.05; Table 1). Moreover, the results indicated no horizontal pleiotropy (the intercept of MR-Egger did not significantly deviate from zero, and *p*-values for an intercept from MR-Egger were > 0.05). No single instrumental variable influenced the estimated causal effects (Figure 4). A directionality examination using Steiger’s analysis did not suggest a violation of causality.

## Discussion

In the past few years, there has been emerging evidence that innate immunity disorders are involved in the occurrence and development of ALS (Chiu et al., 2009; Cirulli et al., 2015; Volpe and Nogueira-Machado, 2015; Oakes et al., 2017). Herein, using a two-sample MR approach, we demonstrated that increased expression levels of CD16^–^CD56^+^ on NK cells and HLA-DR^+^ NK cells were causally associated with a decreased risk of ALS, indicating that these two subsets of NK cells are protective factors against ALS. To the best of our knowledge, this study is the first attempt at elucidating the causal relationship between alterations in NK cells-related immune traits and ALS using a genetic approach based on GWAS summary statistics.

Natural killer cells constitute one of the three major human lymphocyte lineages (T, B, and NK cells), accounting for approximately 5–15% of circulating lymphocytes in healthy adults (Colucci et al., 2003). In addition to being the most important component of innate immunity, NK cells are key players in adaptive immunity (Caligiuri, 2008). Over the past decade, accumulating evidence has revealed that CNS-infiltrating NK cells modulate neuroinflammation, establishing an important role for NK cells in neurodegenerative diseases (Poli et al., 2013; Menees and Lee, 2022; Wang and van de Pavert, 2022). In patients with ALS, NK cells are elevated in the peripheral blood (Gustafson et al., 2017; Murdock et al., 2017). NK cells have also been shown to infiltrate the CNS of both hSOD1^G93A^ mice and patients with ALS (Finkelstein et al., 2011; Garofalo et al., 2020). Recently, IFN-γ-secreting NK cells were shown to colocalize with motor neurons in the spinal cord and motor cortex of hSOD1^G93A^ mice at the early stage of ALS, driving pro-inflammatory microglial activity in the CNS (Garofalo et al., 2020). These findings suggest a potential role and viable therapeutic target for NK cells in ALS.

In clinical practice, a vital problem exists in that the deletion of a classification of immune cells is usually not feasible because it severely disrupts the immune system. To the best of our knowledge, recent studies have only focused on the specific classification of T cells in adaptive immunity in ALS, such as CD4^+^CD25^+^Foxp3^+^ Tregs (Thonhoff et al., 2018). Therefore, probing changes in different subsets of NK cells is critical for a more comprehensive understanding of the peripheral immune abnormalities in ALS. In humans, NK cells exhibit the dynamic expression of surface markers involved in differentiation, trafficking, and cytotoxicity, and different subsets of NK cells possess varying immune functions (O′Brien and Finlay, 2019). In a recent study, changes in trafficking and cytotoxicity markers in NK cells were associated with changes in the revised ALS functional rating scale (ALSFRS-R) (Murdock et al., 2021). Conventionally, according to the differential expression of CD16 and CD56, NK cells in the peripheral blood are classified into two major subsets: CD16^+^CD56^dim^ NK cells and CD16^–^CD56^+^ NK cells (Cooper et al., 2001b; Farag and Caligiuri, 2006). CD16^+^CD56^dim^ NK cells account for approximately 90% of peripheral NK cells and predominantly exert cytotoxic effects. CD16^–^CD56^+^ NK cells only account for approximately 10% of NK cells; however, they play an important role in the regulation of immunity by secreting various cytokines, including IFN-γ, tumor necrosis factor (TNF), and granulocyte colony-stimulating factor (Freud et al., 2017). Accumulating evidence reveals a systematic pro-inflammatory state and the crosstalk between the PIS and CIS in ALS. Elevated levels of blood TNF-α, TNF receptor 1, interleukin 6 (IL-6), IL-1β, IL-8, and vascular endothelial growth factor were observed in patients with ALS compared with those in controls (Hu et al., 2017). As important immunoregulatory and cytokine-secreting cells, CD16^–^CD56^+^ NK cells potentially contribute to the systemic inflammatory state and degeneration of motor neurons by regulating the levels of peripheral cytokines. In a study of multiple sclerosis, IFN-γ^+^ NK cells migrated to the vicinity of the meninges to drive astrocytes to convert to an anti-inflammatory phenotype, reducing the level of neuroinflammation in the spinal cord (Sanmarco et al., 2021). Ziemssen et al. revealed a significantly higher relative amount of CD16^bright^CD56^dim^ NK cells in patients with ALS (Jin et al., 2020). A recent study demonstrated that lower levels of CD56^bright^ NK cells in the CSF were associated with faster progression in ALS patients (Rolfes et al., 2021), thus supporting our results wherein a per standard deviation increase in CD16^–^CD56^+^ NK cells was found to be negatively associated with the risk of ALS.

In addition to classifying NK cells based on T-cell receptor-associated molecules, NK cells can also be subdivided according to other markers, such as NK cell p44-related protein and HLA-DR (a subtype of class II histocompatibility antigens) (Phillips et al., 1984; Vitale et al., 1998). HLA-DR expression in NK cells is a marker of cellular activation. Similar to CD16^–^CD56^+^ NK cells, HLA-DR^+^ NK cells are associated with higher IFN-γ production (Evans et al., 2011; Langers et al., 2014; Erokhina et al., 2018), and IFN-γ induces high HLA-DR expression in NK cells (Yano et al., 1996). In our study, the absolute counts of HLA-DR^+^ exhibited no causal relationship with the risk of ALS; nonetheless, a higher expression level of CD16^–^CD56^+^ was associated with a lower risk of ALS. In fact, a fairly high proportion of CD16^–^CD56^+^ NK cells are also HLA-DR^+^ under normal conditions (Mizrahi et al., 2007). Therefore, we hypothesized that the expression levels of CD16^–^CD56^+^ and HLA-DR^+^ may be related to immune disorders in ALS through the secretion of cytokines, particularly IFN-γ.

The results of our study are reliable, since we enrolled a recently published, high-quality GWAS study on the immune-cells spectrum. Furthermore, the GWAS on ALS selected in the current study was also based on a large sample of the European population. The reliable data sources and study designs provided sufficient statistical power. However, in addition to the limitations of the MR methodology, which have been reviewed previously (Davey Smith and Hemani, 2014), this study has several other limitations: (1) the presently available GWAS data exclusively provided the MFI of CD16^–^CD56^+^ on NK cells; the absolute count of CD16^–^CD56^+^ NK cells is required in future GWAS studies; (2) in this study, the number of SNPs for some NK cells-related immune traits were relatively small (<5) because of the absence of an available corresponding GWAS; and (3) the immune traits that were measured at a specific time point might have been affected by many temporary factors, such as age and lifestyle, which may not reflect the lifelong immune characteristics determined by the encoding gene.

## Conclusion

In conclusion, our MR study suggests that higher expression levels of CD16^–^CD56^+^ on NK cells and HLA-DR^+^ NK cells are associated with a lower risk of ALS. Our work enhances the current understanding of the role of peripheral immune disorders in ALS, especially the role of innate immunity, and provides insight into potential NK cells-based therapeutic approaches in ALS. In the future, more attention should be focused on exploring the potential mechanism of the dysregulation of innate immune-cells subsets in the pathogenesis of ALS, an approach that may provide a theoretical basis for the proposal of new therapeutic strategies.

## Data availability statement

All data in this study were obtained from previously published GWAS studies. Data used can be obtained through cited papers. Further inquiries can be directed to the corresponding author.

## Author contributions

ZG and LB: investigation, methodology, data acquisition and analysis, and visualization. MZ: conceptualization, funding acquisition. LB and MZ: supervision, data curation, reviewing, and editing. All authors contributed to the article, interpretation of the data, drafting of the manuscript, and approved the submission.
