# Supplementary material for: Natural killer cells-related immune traits and amyotrophic lateral sclerosis: A Mendelian randomization study
Source: Front Neurosci. 2022 Sep 29;16:981371. doi: 10.3389/fnins.2022.981371 (PMC9562140; doi:10.3389/fnins.2022.981371)
Supplement: Supplementary file 1 [file Data_Sheet_1.pdf]

Supplementary Table: Summary information for instrumental variables of NK cells-related immune traits

Immune traits	SNP	beta.exposure	pval.exposure	se.exposure	beta.outcome	pval.outcome	se.outcome
NK AC	rs183053322	-0.3926	3.83E-09	0.06648	-0.0766	0.5104	0.1163
	rs7216096	0.2022	6.55E-10	0.03265	-0.0141	0.4329	0.0179
	rs9916257	-0.2005	8.82E-17	0.02398	0.0069	0.6191	0.0139
NK/lymphocytes	rs10512469	0.249	1.98E-15	0.03122	-0.0152	0.3902	0.0177
	rs183053322	-0.408	2.51E-10	0.06431	-0.0766	0.5104	0.1163
	rs9916257	-0.2323	1.51E-23	0.02306	0.0069	0.6191	0.0139
NK/CD3$^-$ lymphocytes	rs11954223	-2.093	9.93E-09	0.3644	0.0492	0.3212	0.0496
	rs12874404	-0.1456	1.60E-08	0.02572	2.00E-04	0.9954	0.0282
	rs183053322	-0.4163	3.35E-10	0.06609	-0.0766	0.5104	0.1163
	rs7218011	0.1822	1.74E-08	0.03225	-0.0124	0.4893	0.0179
	rs9916257	-0.1909	1.21E-15	0.02375	0.0069	0.6191	0.0139
CD16$^-$CD56$^+$ on NK	rs10919544	-0.2555	2.40E-20	0.02745	0.0097	0.533301	0.0156
	rs111579151	-0.5632	4.45E-14	0.07427	-0.0107	0.8048	0.0435
	rs11214436	0.3221	1.59E-28	0.02878	-0.0062	0.660999	0.0141
	rs117533070	0.2856	7.71E-09	0.04932	0.1054	0.0763308	0.0594
	rs148849191	-0.7822	1.11E-10	0.1208	-0.016	0.774801	0.056
	rs17504675	-0.4696	8.59E-10	0.07632	0.0545	0.2669	0.0491
	rs182003559	-0.9029	9.72E-09	0.157	0.0698	0.2257	0.0576
	rs77291736	-1.076	6.93E-155	0.03832	0.0729	0.0958893	0.0438
HLA-DR$^+$ NK AC	rs115805162	0.2631	8.32E-17	0.03144	-0.0082	0.764401	0.0272
	rs71632989	-0.6984	2.05E-136	0.02689	-0.0064	0.7727	0.022
	rs71639910	-0.3019	1.15E-09	0.04947	0.0367	0.3681	0.0408
	rs8025803	-0.2666	1.99E-15	0.03341	-0.0024	0.8796	0.016
HLA-DR$^+$ NK/NK	rs115805162	0.2582	1.32E-15	0.03216	-0.0082	0.764401	0.0272
	rs3933769	0.1574	1.04E-08	0.02744	-0.0385	0.0389897	0.0186
	rs509749	-0.1543	5.41E-09	0.02638	0.0047	0.733599	0.0137
	rs71632989	-0.6848	1.30E-124	0.02772	-0.0064	0.7727	0.022
	rs7180804	-0.2525	8.74E-17	0.03019	0.0012	0.9423	0.0159
	rs865239	-0.1371	4.97E-08	0.02509	0.0072	0.6016	0.0137
	rs9916257	0.1457	6.72E-09	0.02506	0.0069	0.6191	0.0139
HLA-DR$^+$ NK/CD3$^-$ lymphocytes	rs115805162	0.2697	1.62E-17	0.0315	-0.0082	0.764401	0.0272
	rs3933769	0.1562	6.87E-09	0.0269	-0.0385	0.0389897	0.0186
	rs509749	-0.1613	4.80E-10	0.02585	0.0047	0.733599	0.0137
	rs71632979	-0.7124	9.98E-142	0.02686	-0.0072	0.7438	0.022
	rs8025803	-0.2729	4.22E-16	0.0334	-0.0024	0.8796	0.016
CD16$^-$CD56$^+$ on HLA-DR$^+$ NK	rs56199187	0.2389	2.03E-14	0.03108	-0.0051	0.8177	0.022
	rs78358948	-0.3396	1.17E-15	0.04218	0.0712	0.1064	0.0441

Abbreviations: SNP, single nucleotide polymorphism; NK, natural killer cell; HLA, human leucocyte antigen.
