# Supplementary figures and images for: Natural killer cells-related immune traits and amyotrophic lateral sclerosis: A Mendelian randomization study
Source: Front Neurosci. 2022 Sep 29;16:981371. doi: 10.3389/fnins.2022.981371 (PMC9562140; doi:10.3389/fnins.2022.981371)

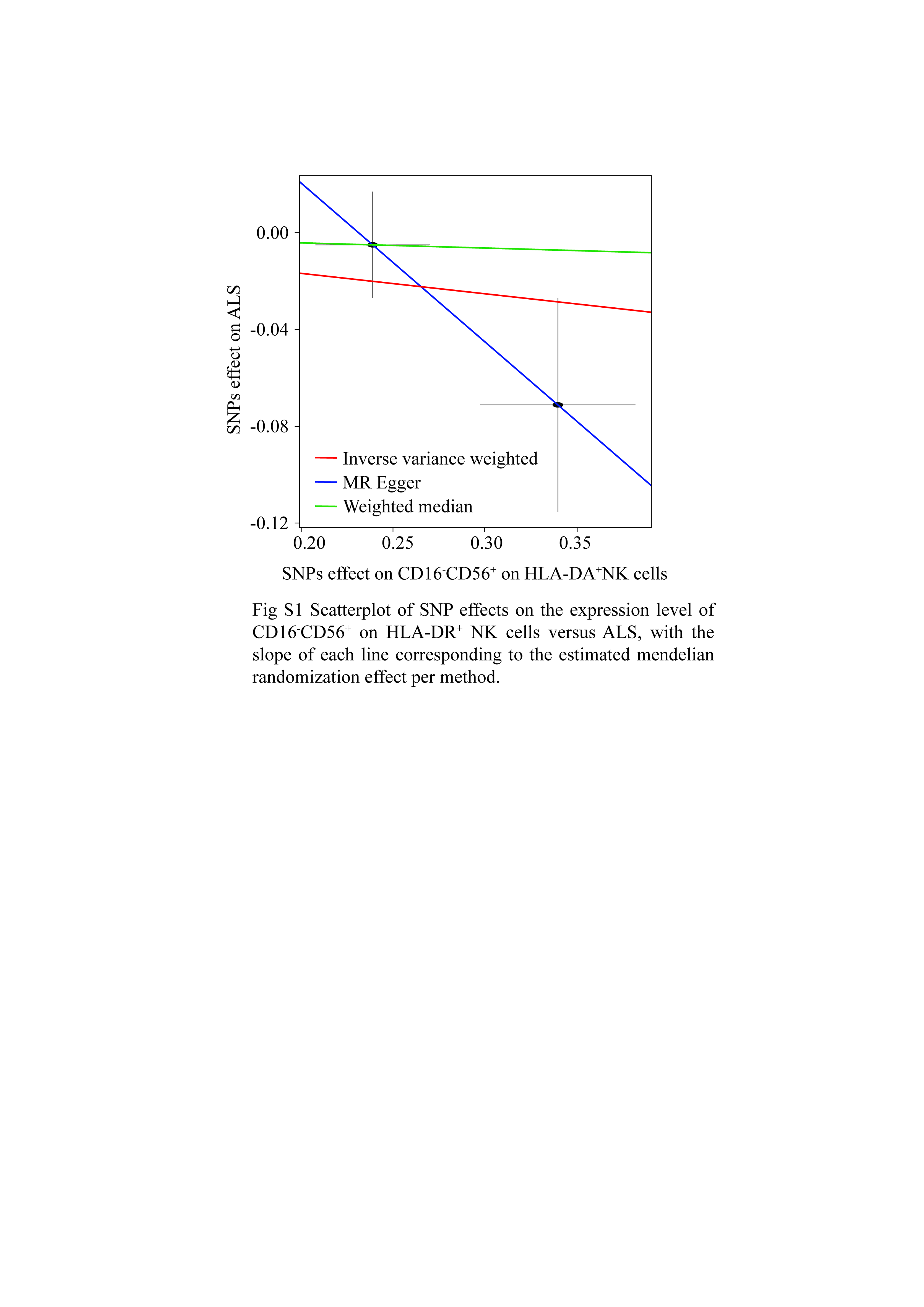

Supplement: Supplementary file 2 [file Image_1.TIF]
